# Bladder p75^NTR^-Mediated Anti-Inflammatory Response via the TLR4/TRAF6/NF-κB Axis

**DOI:** 10.3390/life15060957

**Published:** 2025-06-14

**Authors:** Claudia Covarrubias, Abubakr H. Mossa, Laura R. Yan, Benjamin Desormeau, Philippe G. Cammisotto, H. Uri Saragovi, Lysanne Campeau

**Affiliations:** 1 Lady Davis Institute, McGill University, Montreal, QC H3T 1E2, Canada; claudia.covarrubias@mail.mcgill.ca (C.C.); amossa@sharjah.ac.ae (A.H.M.); laura.yan@mail.mcgill.ca (L.R.Y.); benjamin.desormeau@mail.mcgill.ca (B.D.); philippe.cammisotto.1@ulaval.ca (P.G.C.); uri.saragovi@mcgill.ca (H.U.S.); 2 Urology Department, Jewish General Hospital, Montreal, QC H3T 1E2, Canada

**Keywords:** p75^NTR^, THX-B antagonist, LPS, inflammatory pathways, bladder

## Abstract

Recurrent bacterial cystitis in women can lead to interstitial cystitis or bladder pain syndrome (IC/BPS). Activation of Toll-like receptor 4 (TLR4) by LPS can upregulate signaling of the pro-inflammatory receptor p75^NTR^. The aim of the presented study was to assess whether p75^NTR^ antagonist THX-B can modulate LPS-mediated inflammation in bladder cells. In vitro expression and LPS-activation of p75^NTR^ were confirmed in urothelial (URO) and smooth muscle (SMC) cells. In UROs, p75^NTR^ antagonism abolished the LPS-elicited rise in membrane-bound and soluble TNF-α. However, it could not prevent LPS-induced rise in phosphorylated ERK nor decrease in phosphorylated p38MAPK, nor the increase in iNOS and nitric oxide (NO) content. On the other hand, in SMCs, LPS increased phosphorylation of JNK, nuclear translocation of NF-κB, and association of TRAF6 to p75^NTR^, outcomes prevented by p75^NTR^ antagonism. In UROs, LPS decreased the expression of tight junction proteins, ZO-1 and occludin, with the latter rescued by p75^NTR^ antagonism. Intraurethral instillation of LPS increased inflammation in the lamina propria, activation of JNK, and contractile activity of bladder tissue. Alternatively, intraperitoneal THX-B injections prevented LPS-induced inflammation but not enhanced muscle contraction. Our results suggest that inhibition of p75^NTR^ could help in reducing bladder symptoms during cystitis.

## 1. Introduction

The global burden of bladder infections by *Escherichia coli* (*E. coli*)—hereinafter termed acute bacterial cystitis—has become a serious threat to women’s urologic health worldwide. Every year, 150 million new cases of urinary tract infections (UTIs) are reported in the global female population, prevailing among aging women, of which up to 30% of cases recur within 6 months after treatment [1,2,3]. Bacterial cystitis is experienced, on average, by as many as one in two women at some point in their lifetime [4]. Established risk factors for relapse encompass sexual intercourse, personal hygiene, and lower urinary tract anomalies, though non-communicable diseases such as diabetes and population aging contribute to recurrence due to hormone-related changes [1]. Whereas bacterial cystitis is generally treatable, the emergence of antimicrobial resistance in the past few decades has dramatically skewed the effectiveness of many antibiotics, now mostly inefficient against many strains of uropathogenic bacteria [5].

Women with acute bacterial cystitis manifest symptoms of severe pelvic pain, increased voiding frequency, and/or urinary urgency. Recurrent episodes of bacterial cystitis might contribute to the onset of symptoms of bladder pain syndrome or interstitial cystitis (IC/BPS) [6], possibly leading to progression of chronic features of bladder inflammation [7]. However, the exact causal mechanisms have yet to be clearly identified to develop new effective therapeutics [8].

Nevertheless, several comprehensive studies on voiding dysfunction have stressed the presence of the Toll-like receptor 4 (TLR4) and the p75 neurotrophin receptor (p75^NTR^) in bladder tissue [9,10]. These transmembrane receptors bind, respectively, *E*. *coli* lipopolysaccharide (LPS) and a variety of neurotrophic factors. Among these, the proform of the nerve growth factor (proNGF) and brain-derived neurotrophic factor (proBDNF) synthesized by neurons, mastocytes, urothelial (URO), and smooth muscle cells (SMCs) were found to have the highest inflammatory protein profile in injured organs during diabetes and spinal cord injury [11,12]. TLR4 receptor intracellular pathways include binding of MyD88 and IRAK proteins, triggering through TRAF6 the activation of p38MAPK, ERK, and JNK pathways with activation of genes controlled by NF-kB and CREB, leading to the release of pro-inflammatory cytokines [13]. Receptor p75^NTR^, after binding proneurotrophins, binds TRAF6, leading to increases in the phosphorylation of JNK, activation of caspases, and nuclear translocation of NFkB [14].

The similarity in inflammatory and cell degenerative responses elicited by LPS/TLR4 and proNGF/p75^NTR^ signal transduction cascades suggested the possibility of an interplay between both receptors, reinforcing the progression of chronic inflammation [15,16]. This crosstalk between receptors might involve similar pathways, or activation or inhibition of their respective cascade that might be mediated by direct interaction between intracellular proteins or indirect influences mediated by shared downstream proteins.

Blocking of proNGF/p75^NTR^ signaling can be achieved with either an anti-proNGF monoclonal antibody or a highly specific pharmacological antagonist of p75^NTR^, THX-B. The latter is a small molecule, a synthetic chemical with the following formula: (1,3-diisopropyl-1-[2-(1,3-dimethyl-2,6-dioxo-1,2,3,6-tetrahydro-purin-7-yl)-acetyl]-urea). It is a competitive inhibitor blocking the binding of proneurotrophins to receptor p75^NTR^. Its use has so far been limited to basic scientific studies and is usually injected intraperitoneally in vivo in rodents, where it remains active for a week. For example, THX-B decreased inflammation in diabetic retina and traumatic brain injury [17,18], and reduced proNGF bladder content independently of hyperglycemia in diabetic mice [10]. In this perspective, the present study examines whether p75^NTR^ antagonist THX-B could decrease inflammatory processes elicited by LPS, in vitro on primary cultures of URO and SMC, and in vivo on C57 mice bladder tissue after intraurethral instillation of LPS from *E. coli*.

## 2. Materials and Methods

### 2.1. Animals

McGill University Animal Ethics Committee (Montreal, QC, Canada) approved all in vivo experiments. Standards of the Canadian Council for Animal Care (CCAC) were thoroughly followed. Female rodents (Sprague-Dawley rats and C57Bl6 mice) aged 8-week-old (Charles River Laboratories, QC, Canada) were housed in individual cages with free access to water and food (standard Purina chow, Teklad Global, Madison, WI, USA) ad libitum, and kept on a 12 h light/dark cycle.

### 2.2. Cell Culture

Female Sprague Dawley (SD) rats were exsanguinated by cardiac blood withdrawal under anesthesia (isoflurane 3%) and sacrificed. Bladders were excised and placed in cold sterile phosphate-buffered saline (PBS) (pH 7.4). Urothelium layer was carefully scraped and left for 15–20 min in Dulbecco’s modified Eagle medium (DMEM) containing 100 U/mL of collagenase type IV at 37 °C, as previously reported [19]. Cells were then washed twice in DMEM containing 10% fetal bovine serum (FBS) then seeded and kept at 37 °C in a humidified incubator with 5% CO_2_ atmosphere in Dulbecco’s DMEM low glucose/keratinocyte (50/50) medium containing FBS (10%), GlutaMAX (X1), a mix of growth factors (insulin 5 µg/mL, dihydrocortisone 0.5 µg/mL, adenine 15 µg/mL, ethanolamine 0.1 mM), Rho inhibitor 4-((1R)-1-aminoethyl)-N-pyridin-4-ylcyclohexane-1-carboxamide (Y27623) (10 µM), and 1% penicillin/streptomycin (100 U/mL, 100 µg/mL). The medium was changed every 2 to 3 days until cell confluency. Cells were starved for 24 h in DMEM/MCDB153 medium without Y27623 prior to use.

In parallel, detrusor muscles were finely minced and incubated for 45 min with intense shaking in DMEM containing 250 U/mL of collagenase type IV. Undigested tissues were removed with a strainer (40 µm mesh) and primary SMCs were washed twice in DMEM/FBS (10%) and cultured in similar conditions to urothelial cells in Petri dish containing DMEM supplemented with FBS (10%), high glucose (27 mM) and penicillin/streptomycin (100 U/mL, 100 µg/mL). Prior to use, cells were starved for 72 h in normoglycemic medium.

### 2.3. In Vivo Transurethral Infection

Microtubes (0.011″ l.D × 0.024″ O.D., Braintree Scientific, MA, USA) were sterilized in a Cidex OPA bactericidal solution (Johnson&Johnson, Markham, Canada, ON) for 30 min, then washed in sterile PBS. LPS from *E. coli* (O55:B5, Sigma-Aldrich, St-Louis, MO, USA) was dissolved in sterile PBS (1 mg/mL). C57 mice were anesthetized with isoflurane and injected intraperitoneally with 100 μL saline or THX-B (50 μg/mouse in PBS) or/and inserted via the urethra with 100 μL of PBS or LPS solution (100 μg per mouse). The groups were allocated to the following groups: (1) PBS-injected/PBS-inserted (CTL), (2) THX-B injected/PBS-inserted (THX-B), (3) PBS-injected/LPS-inserted (LPS), and (4) THX-B injected/LPS-inserted (LPS+THX-B). For urethral insertion, bladders were emptied by gentle massage on the belly and the urethral meatus cleaned with sterile gauze and chlorhexidine gluconate (Baxedin, Omega Laboratories, Montreal, QC, Canada). Urethral catheters were lubricated with a water-based lubricant (Vaseline, Unilever Canada, Toronto, ON, Canada), then transurethral catheterization was performed. Bladders were slowly instilled with 100 μL saline or LPS. The catheters were secured with adhesive and maintained for 1 h. Mice were then kept 24 h post-surgery. After cardiac blood withdrawal under isoflurane anesthesia, urinary bladders were removed, placed in cold PBS, and processed for organ bath, immunoblotting, and histology.

### 2.4. Bladder Physiological Recordings

The bladder dome and base were removed, and two transversal strips were obtained. The remaining tissue was kept for protein extraction and histology. Strips were mounted in a 4-channel Tissue Bath System (720 MO, DMT Inc., Ann Arbor, MI, USA) in wells containing 6 mL of Krebs–Ringer bicarbonate solution pH 7.4, with constant oxygenation (95% O_2_, 5% CO_2_) at 37 °C. Basal muscle tension was kept at 0.5 g. The solution was changed every 15 min. Strips were successively stimulated with KCl (60 mM) and carbachol (3 nM to 100 μM). Electrical field stimulation (EFS; 1–32 Hz) was performed using a Grass Technologies S88 Stimulator (West Warwick, RI, USA). Tension values were normalized to the weight of bladder strips and analyzed with the LabChart 7 software (ADInstrument, Colorado Springs, CO, USA).

### 2.5. Western Blotting

Cell cultures and bladder pieces were homogenized in ice-cold RIPA buffer containing an antiprotease mix (Roche Diagnostics, Montreal, QC, Canada). Protein concentrations were measured with a MicroBCA assay kit (Boster Biological Technology, Pleasanton, CA, USA). Equal amounts of proteins (15–30 µg) were resolved on a 6–8% SDS-PAGE polyacrylamide gel and then transferred to PVDF membranes. After blocking (TBS-Tween 0.1% with non-fat milk 5%) for 1 h, overnight incubation was carried out at 4 °C with the following primary antibodies: inducible nitric oxide synthase (iNOS) (1/4000), extracellular signal-regulated kinase (ERK) (1:1000), P-ERK (1:1000), c-Jun N-terminal kinase (JNK) (1:1000), P-JNK (1:1000), P38 mitogen-activated protein kinase (p38MAPK) (1:2000), P-p38MAPK (1:2000), Nuclear factor kappa B (NF-κB) p65v (1:1000), TNF-α (1:2000), lamin B1 (1:2000), ß-actin (1:20000), tumor necrosis factor receptor-associated factor 6 (TRAF6) (1:600), occludin (1:8000), E-cadherin (1:2000), ZO-1 (1:2000), smoothelin (1:2000) and p75^NTR^ (1/8000). After thorough washings, membranes were incubated with a secondary HRP-bound antibody (1:3000) (Millipore, San Diego, CA, USA) for 1 h. Signals were revealed with Luminata Crescendo HRP substrate (Millipore, Billerica, MA, USA) and quantified using ImageJ v1.50i.

### 2.6. RTqPCR

Total RNA was extracted using a phenol–chloroform protocol and quantified using a nanodrop system. Primers were obtained from Integrated DNA Technologies (IDT, Coralville, IA, USA): p75^NTR^ forward 5′-GAGGGCACATACTCAGACGA-3′, p75^NTR^ reverse 5′-CTCTTCGCATTCAGCATCAG-3′, GAPDH forward 5′-TGC CAC TCA GAA GAC TGT GG-3′, GAPDH reverse 5′-TTC AGC TCT GGG ATG ACC TT-3′. Sensifast Low-ROX kit containing SYBR-green was used with an Applied Bioscience 7500 Fast Real-Time PCR (RT-qPCR). Amplification conditions were as follows: at 95 °C for 10 min, then 45 cycles of 95 °C for 15 seconds and 57 °C for 40 s, with melt curve analysis. All samples were performed in triplicate. Each primer was controlled for specificity and efficiency (90–110%). Controls were carried out using purified RNA without reverse transcription. Data were analyzed using the 2^−ΔΔCT^ method [20].

### 2.7. NF-kB Nuclear Translocation

Nuclear content in NF-κB was assessed as previously described [21]. Briefly, cells were incubated with LPS (100 ng/mL) from *E. coli* for 30 min. The supernatant was discarded, and cells were washed with ice-cold PBS, then homogenized in a Tris-NaCl buffer (20 mM Tris pH 8, 100 nM NaCl, 300 mM sucrose, 3 mM MgCl_2,_ containing antiprotease cocktail from Roche Diagnostics). After 10 min on ice, the homogenates were centrifuged at 3000 rpm for 10 min at 4 °C. The supernatants corresponding to the cytoplasmic fraction were used for the measurement of proteins, while the pellets (nuclear fraction) were homogenized in another specific buffer (20 mM Tris pH 8.0, 300 mM NaCl, 2 mM EDTA pH 8.0, with antiprotease cocktail). After 30 min on ice, homogenates were centrifuged at 24,000 rpm for 20 min at 4 °C. Supernatants were used to measure nuclear proteins. Both fractions (cytoplasmic and nuclear) were analyzed by Western blotting as described for the semi-quantification of NF-κBp65, lamin B1, and ß-actin.

### 2.8. Immunoprecipitation

Cells were lysed with RIPA buffer containing antiprotease mix and left on ice for 10 min. Equal amounts of protein (200–400 µg) were immunoprecipitated overnight with anti-p75^ICD^ rabbit antibody (1:600) at 4 °C with gentle rocking. The following morning, 20 µL of protein G agarose beads (ThermoFisher, Montreal, QC, Canada) were added and left for 3 h at 4 °C. After centrifugation (7000 rpm, 3 min), pellets were washed 5 times with ice-cold 1X cell lysis buffer, then resuspended and processed for SDS-PAGE gel.

### 2.9. Histology

Pieces of bladder were successively immersed in 10, 20, and 30% sucrose in PBS, 24 h at each concentration. Tissues were then bathed in OCT (Leica Biosystems, Lincolnshire, IL, USA) and placed at −80 °C for 1 h, then kept frozen on dry ice. Tissue sections were cut (7 μm thickness) using a Leica CM3050S cryostat. Staining with Hematoxylin and Eosin (H&E) was performed following a standard protocol from the manufacturer (Abcam, Cambridge, MA, USA). Bladder wall and compartment thickness were measured using an LSM800 microscope (Carl Zeiss Canada, Toronto, ON, Canada). Immune cells, including lymphocytes, granulocytes, and basophils were counted on the same slides and results reported as the number of immune cells per square millimeter of lamina propria.

### 2.10. Immunohistochemistry

Cells were seeded on glass coverslips until confluency, then fixed for 30 min in 4% paraformaldehyde in PBS pH 7.4. Coverslips were washed with PBS and cells permeabilized with Triton X100 (0.1% in PBS, pH 8.0). After washing in PBS, blocking was performed with BSA 1% in PBS for 1 h. The cells were incubated with primary antibodies overnight at 4 °C. Incubation with the secondary antibody conjugated to Dylight488 (Thermo Fisher Scientific, St-Laurent, QC, Canada), for 1 h was followed by thorough washing with 1X Tris-buffered saline containing 0.1% Tween-20 (TBST). Slides were finally mounted on DAPI for examination under fluorescence microscopy (Leica Microsystem, Lincolnshire, IL, USA).

### 2.11. Nitric Oxide Assay

Levels of NO were measured in cell culture media using the Griess colorimetric method involving sulfaminalide/N-(1-naphthyl)ethylenediamine dihydrochloride (NEDD) [22].

### 2.12. Elisa Kit

NGF, proNGF, and p75^ECD^ were measured using kits from Biosensis (Thebarton, Australia). TNF-α content in bladder homogenates was assessed with a kit from BosterBio (Pleasanton, CA, USA).

### 2.13. Data Analysis

Data presented in this study were obtained from May 2018 to April 2021. Values were expressed as mean ± S.E.M. Statistical significance was established as * *p* < 0.05, ** *p* < 0.01, and *** *p* < 0.001. GraphPad Prism Software (Version 10.4.1) was used to compare differences between control, LPS, and THX-B groups by performing unpaired Student’s *t*-test and One-way ANOVA (Bonferroni post hoc test). EC_50_ values were determined using the extra sum-of-squares F-test for non-linear regression.

## 3. Results

### 3.1. Expression and Activation of Receptor p75^NTR^ in Bladder Cells

**Figure 1:**
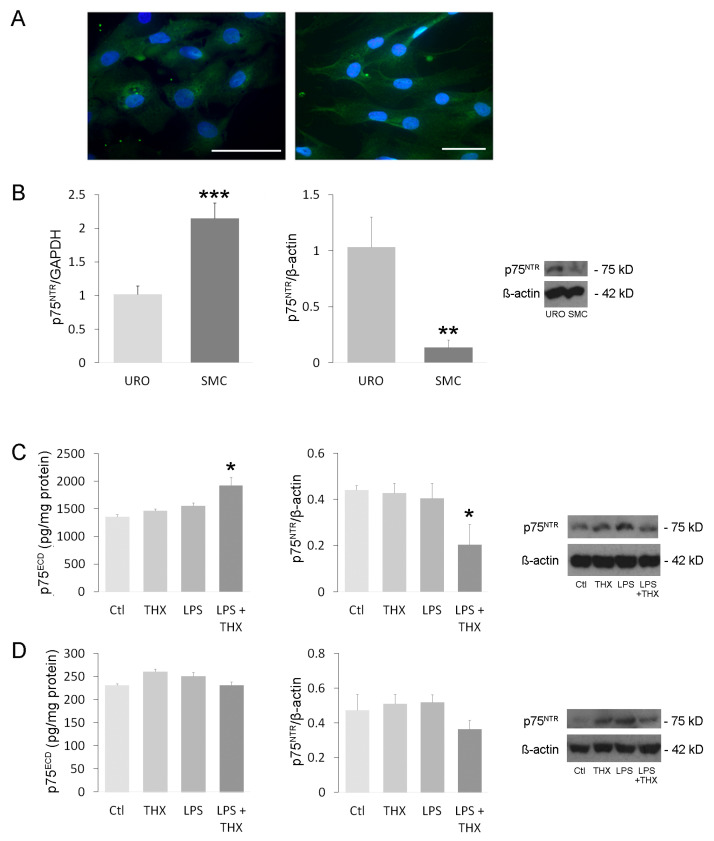
Expression of p75^NTR^ in urothelial (URO) and smooth muscle (SMC) cells. (**A**) Immunochemistry revealed p75^NTR^ in the cytoplasm and plasma membranes of UROs (**left**) and SMCs (**right**) (Bars = 25 μm). (**B**) Relative expression of p75^NTR^ mRNA and semi-quantification of p75^NTR^ proteins in URO and SMC extracts were carried out (Student *t*-test, *n* = 6, ** *p* < 0.01, *** *p* < 0.005). In UROs (**C**) and SMCs (**D**), extracellular p75^ECD^ and intracellular p75^NTR^ were measured after a 24 h incubation in the presence of LPS (100 ng/mL) and/or THX (5 μg/mL) (*n* = 6). One-way ANOVA, * *p* < 0.05.

In order to confirm the presence of receptor p75^NTR^ in UROs and SMCs, immunohistochemistry, RTqPCR, and immunoblotting were carried out. Immunostaining confirmed the presence of p75^NTR^ in URO and SMC cultures, at the levels of the plasma membrane and in the cytoplasm (Figure 1A). RTqPCR found higher mRNA expression of the receptor in SMCs, while immunoblotting detected more of its proteins in UROs (Figure 1B). P75^ECD^, the extracellular domain of p75^NTR^ released into the medium after activation of the receptor, was accordingly six times higher in supernatant from URO (Figure 1C) than from SMC (Figure 1D) in basal (control) conditions. Incubation of cells with LPS (100 ng/mL) or THX-B (5 µg/mL) for 24 h did not affect the cleavage of p75^NTR^. However, combination of both components resulted in the increase of p75^ECD^ in the medium of UROs with concomitant decrease in membrane-bound receptor (Figure 1C). SMCs were unaffected by either of the two compounds (Figure 1D). Finally, when exposed to growing concentrations of LPS, we did not observe a change in the protein expression of p75^NTR^ intracellular and extracellular domains in either URO or SMCs, suggesting that cell sensitization to proNGF/p75^NTR^ inflammatory activity is not dependent on TLR4 signals in bladder cells.

### 3.2. TNF-α Cell Content and Nitric Oxide (NO) Secretion in the Presence of LPS and THX-B

**Figure 2:**
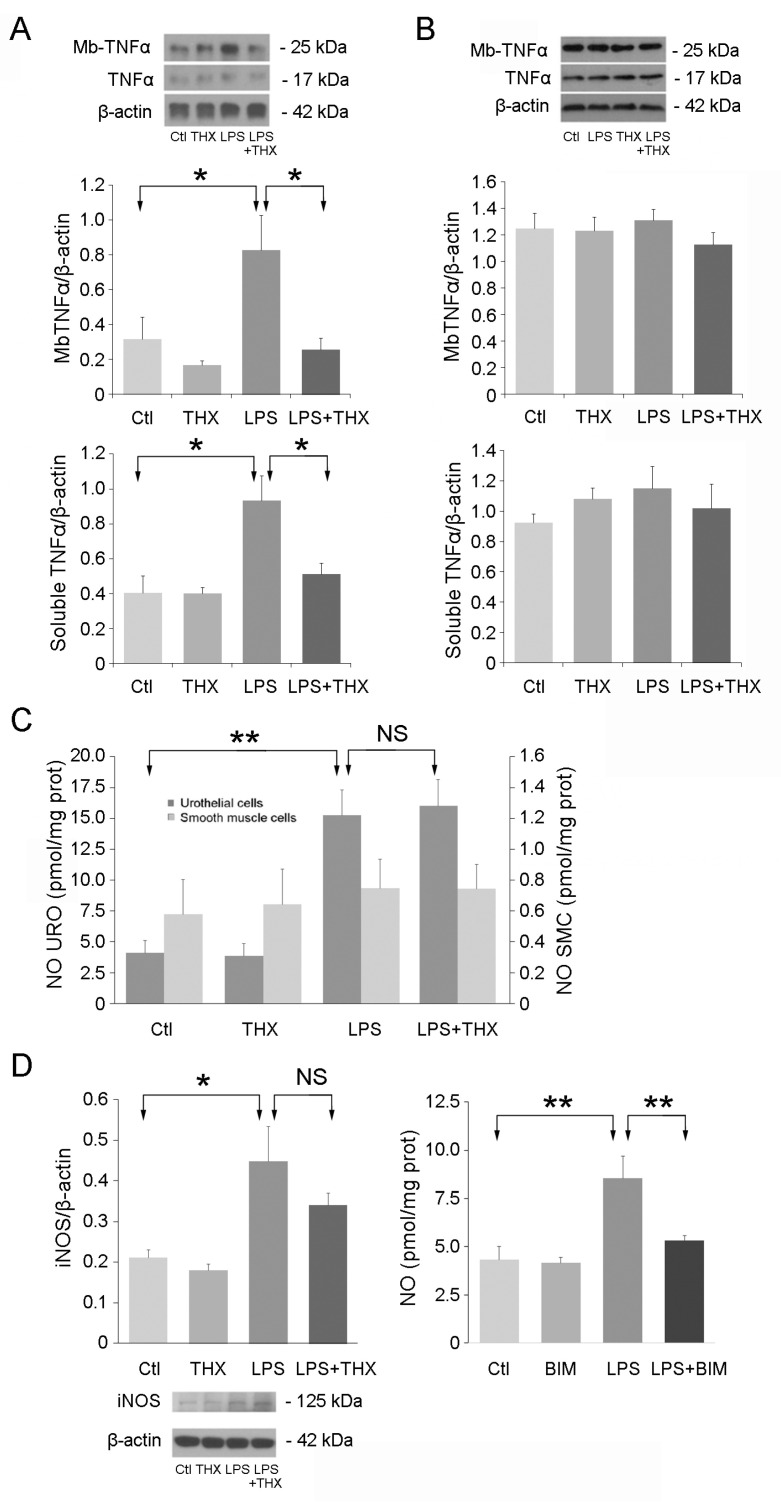
TNF-α and NO synthesis in the presence of LPS and THX-B. (**A**) URO and (**B**) SMC were incubated with LPS (100 ng/mL) alone or combined with THX-B (5 μg/mL) for 24 h. The membrane-bound and soluble forms of TNF-α were semi-quantified by immunoblotting (*n* = 5). (**C**) In the same samples, NO levels in culture medium from UROs and SMCs were measured (*n* = 7). (**D**) In URO cell extracts, iNOS was semi-quantified by immunoblotting. Extracellular NO was measured after 24 h with the PKC inhibitor BIM (100 μM) (*n* = 5). One-way ANOVA, * *p* < 0.05, ** *p* < 0.01.

LPS is known to increase the synthesis and secretion of TNF-α and NO in IC/BPS [23]. TNF-α is a cytokine released by immune and non-immune cells in response to tissue injury and contributes to urothelial antimicrobial resistance to pathogenic bacteria. NO is another mediator of inflammation produced by the iNOS enzyme that participates in nociception and SMC relaxation [24]. Inoculation of *E. coli* bacteria or instillation of LPS only in mice and rat bladders enhance iNOS expression in the urothelium [25]. To verify whether these processes also occurred in UROs and detrusor SMCs in culture, cells were incubated for 24 h with LPS (100 ng/mL) with or without pretreatment with THX-B (5 µg/mL). In UROs, levels of membrane-bound TNF-α and soluble TNF-α were increased by LPS and abolished by THX-B, suggesting that p75^NTR^ receptor can counteract the signaling of TLR receptors (Figure 2A). No changes could be seen in smooth muscle cells (Figure 2B). On the other hand, in the same conditions, NO and iNOS were enhanced by LPS in urothelial cells, with no effect of preincubation with THX-B (Figure 2C), suggesting that the stimulation of TNF-α and iNOS uses different pathways [23]. This point was indeed confirmed by adding bisindolylmaleimide (100 µM) to the medium, an inhibitor of the TLR4 s messenger PKC [26], resulting in decreased LPS-elicited NO secretion in urothelial cells (Figure 2C).

### 3.3. Inflammatory and Survival Pathways in Bladder Cells During LPS Incubation

**Figure 3:**
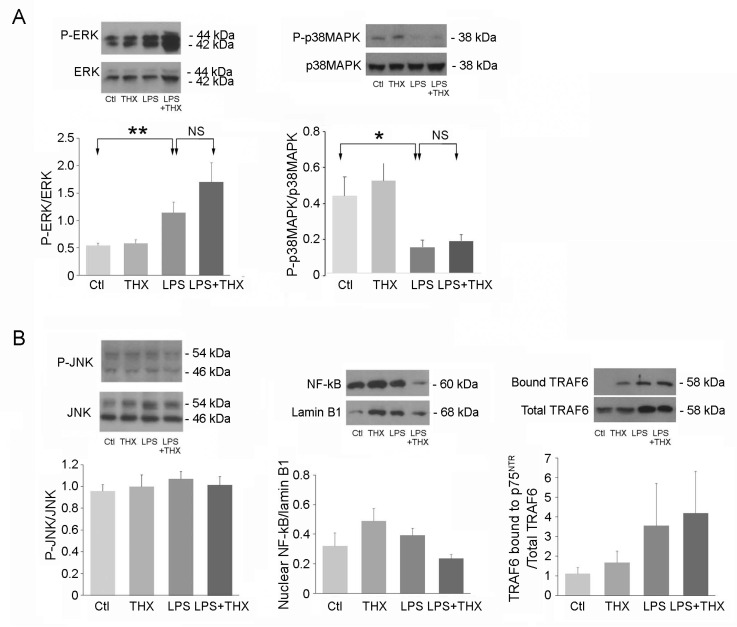
Intracellular pathways in URO. (**A**) Cells were preincubated with or without THX-B (5 μg/mL) then exposed to LPS (100 ng/mL) for a short time as follows: 2 min for assessment of ERK activation, 30 min for p38MAPK (*n* = 5), and (**B**) 10 min for JNK and 5 min for translocation of NF-κB into the nucleus (*n* = 9) and association of TRAF6 to p75^NTR^ (*n* = 5). One-way ANOVA, ** *p* < 0.01, * *p* < 0.05.

An overwhelming number of studies observed closely identical biological responses by TLR4 and p75^NTR^ after exposure to their respective ligands LPS and proNGF; both receptors are associated with similar intracellular pathways, the most important being ERK (survival) and JNK (inflammatory) [15,16]. In particular, TLR4 and p75^NTR^ intracellular domains have the ability to recruit the TRAF6 ubiquitinase as a signaling intermediate, conveying NF-κB and JNK signals [27]. LPS (100 ng/mL) or THX-B (5 µg/mL) was added individually and combined in URO and SMC cultures. In UROs, LPS triggered an increase in the phosphorylation of ERK and a decrease in p38MAPK (Figure 3A) that were unaffected by THX-B, suggesting that LPS affects ERK and p38MAPK pathways independently of p75^NTR^. In contrast, the ratio of p75^NTR^-associated with TRAF6 to total TRAF6 was unaffected by LPS or THX-B in short-term incubation (5 min) (Figure 3B). In accordance, levels of activated JNK and NF-κB translocation were not different between control and LPS-incubated cells after 10 and 30 min of incubation and remained stable in the presence of THX-B (Figure 3B).

**Figure 4:**
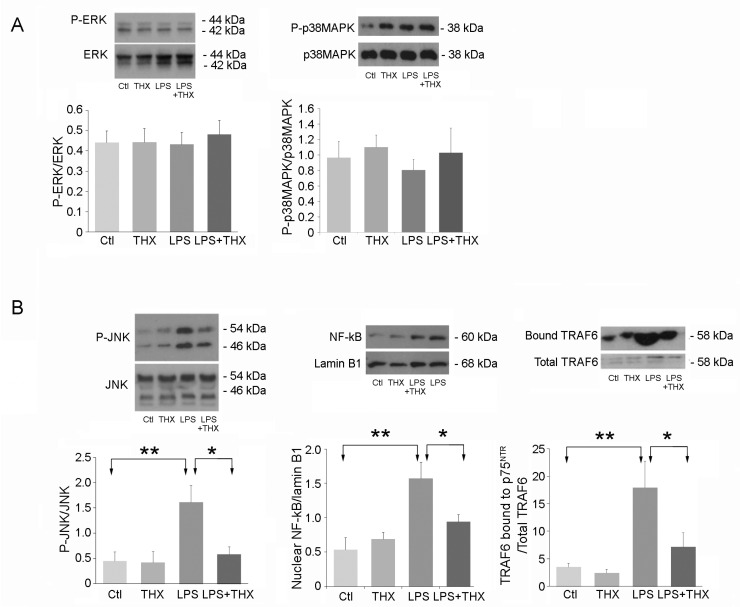
Intracellular pathways in SMC. (**A**) Exposure of cells to LPS (100 ng/mL) with or without pre-incubation with THX-B (5 μg/mL) was performed to detect activation of ERK (*n* = 6) and p38-MAPK (*n* = 5). (**B**) JNK activation, NF-κB translocation into the nucleus (*n* = 9), and association of TRAF6 to p75^NTR^ (*n* = 6) were carried out in parallel, in the same incubation conditions described for URO. One-way ANOVA, ** *p* < 0.01, * *p* < 0.05.

On the other hand, in SMCs, neither ERK nor p38MAPK activation was affected by LPS or THX-B (Figure 4A). However, JNK phosphorylation, association of TRAF6 on p75^NTR,^ and translocation of NF-κB were potently stimulated by LPS and prevented by preincubation with THX-B (Figure 4B). To determine the resulting action of these pathways in UROs and SMCs, we tested the initiation of caspase-3 activity as one of the main steps in apoptosis, and caspase-8 activity as a pro-inflammatory and pro-apoptotic mediator [28]. These two processes are activated by the JNK/NF-κB pathway. No elevation of caspase activity was detected following 24 h incubation with LPS (100 µg/mL) (unpublished observation).

### 3.4. LPS on Tight Junctions

**Figure 5:**
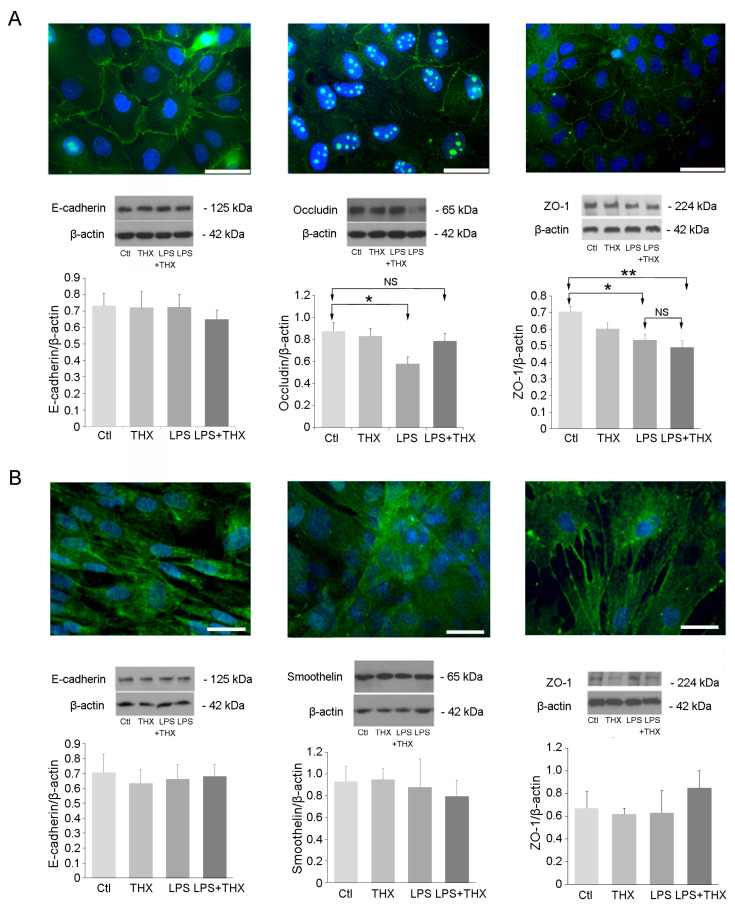
Tight junction and contractile protein levels after LPS and THX-B addition. (**A**) Immunohistochemistry detected E-Cadherin, Occludin, and ZO-1 expression on the URO membrane and inside the cytoplasm. Quantification of these proteins was performed after 24 h exposure to LPS (100 ng/mL) with or without THX-B (5 μg/mL). Bars = 50 µm. (*n* = 6), One-way ANOVA, * *p* < 0.05, ** *p* < 0.01. (**B**) Detection of E-cadherin, smoothelin, and ZO-1 was performed on SMCs. Immunoblotting was carried out after a 24 h incubation with LPS or THX-B for all proteins (*n* = 6). Bars = 25 µm.

Tight junctions are expressed by bladder cells and are known to be affected during inflammation [29,30]. Occludin and Zonula Occludens 1 (ZO-1) are proteins of the intercellular tight junctions, providing structural integrity of epithelia to create a polarized barrier to prevent passage of substances through the paracellular space [31]. E-cadherin is an adhesion molecule bound to tight junction proteins and the actin cytoskeleton [32]. Smoothelin is a contractile protein part of the cytoskeleton and specific to muscle cells [33]. LPS (100 ng/mL) was added individually or in combination with THX-B (5 µg/mL) to the culture media of UROs or SMCs for 24 h. In UROs, three tight junction proteins were revealed by immunostaining, namely E-cadherin, occludin, and ZO-1 (Figure 5A). Staining of these junctions was observed on the plasma membrane as well as in the cytoplasm (Figure 5A). LPS did not alter E-cadherin protein levels but downregulated occludin and ZO-1 cell content. (Figure 5A). Pre-treatment with THX-B could not rescue the decline in ZO-1 but completely restored occludin levels (Figure 5A). In SMC, E-cadherin, ZO-1, and smoothelin were all unaffected by LPS or THX-B (Figure 5B).

### 3.5. Assessment of In Vivo Inflammation

**Figure 6:**
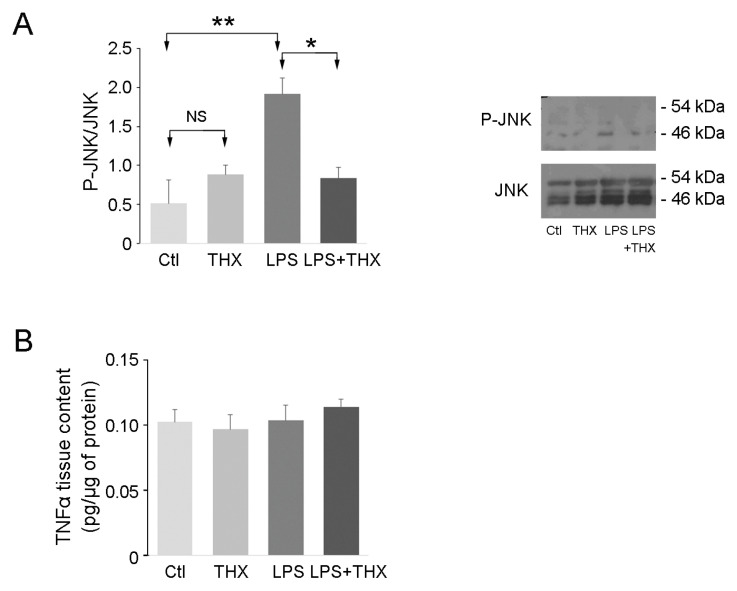
JNK and TNF-α in bladder tissue extracts. (**A**) Activation of JNK and (**B**) levels of TNF-α were measured in bladder extracts from treated and untreated mice after 24 h. (*n* = 6–8), one-way ANOVA compared to controls, * *p* < 0.05, ** *p* < 0.01.

**Figure 7:**
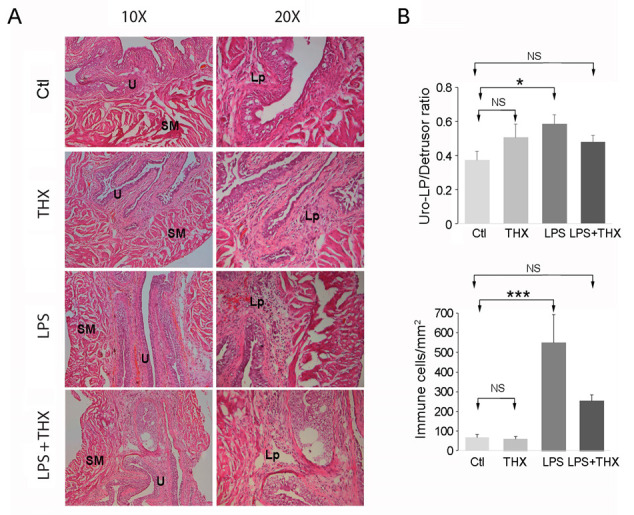
Bladder tissue histology of treated and untreated mice. (**A**) Hematoxylin–eosin staining was carried out on bladder samples from the four groups of mice (Ctl, THX-B, LPS, and LPS+THX). Legend: (U) urothelium, smooth muscle (SM), Lamina propria (Lp). (**B**) The ratio of urothelium (Uro)-lamina propria (LP)/detrusor and immune cell density in the lamina propria was measured. (*n* = 4), ANOVA one-way * *p* < 0.05, *** *p* < 0.005.

To examine the in vivo effect of THX-B on LPS-induced inflammation in bladder tissue, C57 mice were divided into four groups. The controls received an intraperitoneal (i.p) injection of sterile PBS (100 µL). The THX-B-treated group was injected i.p at a concentration of 50 µg/mouse. Finally, LPS (100 µg) in 100 µL was inserted through the urethra in half of the PBS and THX-B treated groups, as described in the Materials and Methods Section. After 24 h, the mice were sacrificed, and their bladders examined for inflammatory markers. Activation of JNK was confirmed by immunoblotting in the LPS group and completely reversed by treatment with THX-B (Figure 6A). Bladder content in TNF-α was unchanged in all conditions (Figure 6B), which might be explained by the dilution of TNF-α originating from urothelial cells in bladder homogenates. Histological analysis using hematoxylin–eosin staining revealed an increase in the ratio of urothelium/lamina propria to detrusor and an increase in the density of immune cells by LPS (Figure 7A,B). Both were prevented by treatment with THX-B (Figure 7A,B).

### 3.6. Organ Bath Recordings

**Figure 8:**
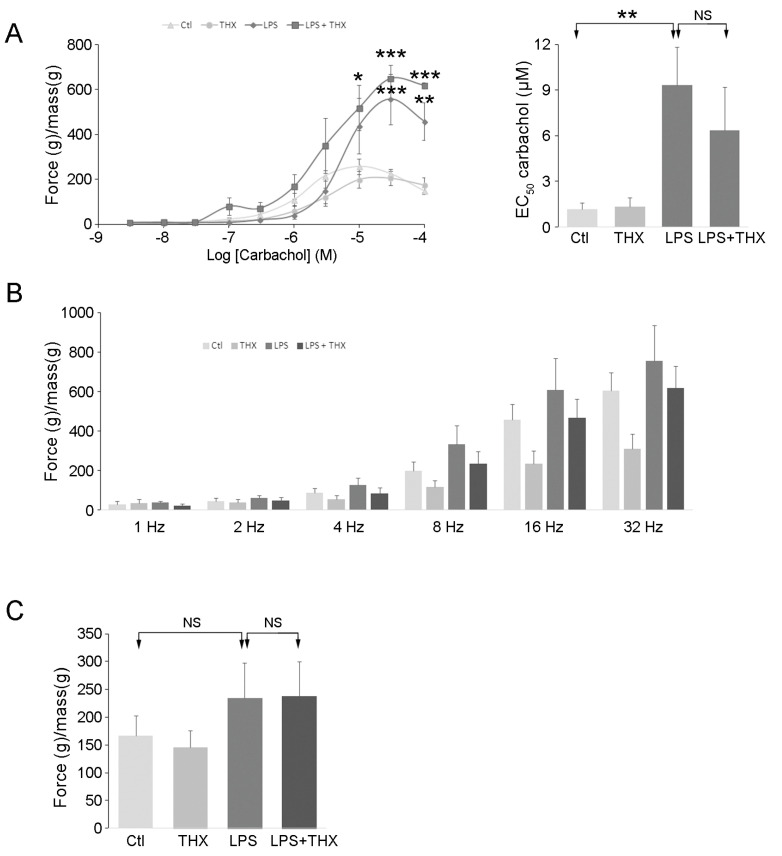
Contractile properties of bladder strips from mice treated with THX and/or LPS. (**A**) Bladder strips from urethral PBS-inserted, without (CTL) or with intraperitoneal injection of THX-B (50 μg) (THX), and from urethral LPS (100 μg) inserted without (LPS) or with intraperitoneal injection of THX-B (50 μg) (LPS+THX) mice were incubated in an organ bath in the presence of increasing concentrations of carbachol (from 3 nM to 100 μM). EC_50_ for carbachol was computed for each group. (**B**) Stimulations by electric field (EFS) (from 1 Hz to 32 Hz) and (**C**) KCl (60 mM) were carried out on the same tissues. Mean ± SEM, (*n* = 6), One-way ANOVA compared to controls, * *p* < 0.05, ** *p* < 0.01, *** *p* < 0.001.

Cystitis triggers inflammation and irritation of the bladder wall, leading to increased bladder contraction [7]. To assess how LPS affects bladder contraction in vivo, bladder strips from the same previously described groups of mice were pinned in organ bath wells containing Krebs–Ringer bicarbonate buffer at 37 °C under oxygen/carbon dioxide bubbling. Increasing concentrations of carbachol were added to stimulate contractions. Higher contractile force was observed in the group treated with LPS compared to controls (PBS-treated), while THX-B had no effect on either (Figure 8A). EC_50_ computation confirmed these results (Figure 8A). EFS did not reveal any significant changes between groups (Figure 8B), nor did KCl (60 mM) stimulation (Figure 8C). These data suggest that only purinergic and muscarinic receptors were affected by LPS in vivo, independently of THX-B.

## 4. Discussion

The presented study aimed to explore the crosstalk between TLR4, the receptor of LPS, and p75^NTR^, the receptor of neurotrophins and proneurotrophins, in modulating the inflammatory response in bladder cells. Both receptors engage a network of cell-type-specific intracellular pathways. We here report that THX-B, a highly specific antagonist of p75^NTR^, had a beneficial effect on bladder tissue, by decreasing the secretion of pro-inflammatory factors in urothelial cells and by preventing activation of the pro-inflammatory pathway JNK/NF-κB in SMCs.

Neurotrophins and their precursors are synthesized and released by bladder cells and act in a paracrine fashion on their membrane-bound receptor TrkA and p75^NTR^ [34,35]. We confirmed and compared the expression of p75^NTR^ between UROs and SMCs. Higher levels of the mature protein were found in UROs, which reflected the remaining amount of p75^NTR^ after LPS and THX-B treatment, with a decrease in membrane-bound receptor and an increase in p75^ECD^, only in UROs. These observations are due to the cleavage of the full-length receptor after activation [36]. Increases in p75^ECD^ were reported to be beneficial and associated with decreased apoptosis of sympathetic neurons during oxidative stress [37].

ProNGF and proBDNF are both ligands of p75^NTR^. We observed an increase in proNGF release elicited by LPS in urothelial cells but not in SMC (preliminary unpublished observations). LPS-stimulated release of proNGF has been well documented for microglial cells in their role in neuroinflammation [38]. On the other hand, proBDNF in the same cell extracts did not display any changes. UROs and SMCs are known to activate a range of intracellular pathways specific to each cell type [21,39]. Activation of p75^NTR^ is also known to be quite complex, as the receptor exists in several oligomerization states that influence its level of activation [40]. We suggest that the differences observed in the levels of p75^NTR^ and p75^ECD^ after LPS stimulation result from increased secretion of proNGF or other proneurotrophins that, in turn, act in a paracrine fashion on p75^NTR^ in urothelial cells, while basal activation of the receptor in SMCs is affected by THX-B.

The activation of p75^NTR^ by proneurotrophins increases the secretion of pro-inflammatory factors, including TNF-α and nitric oxide [23,41]. ProNGF, in particular, increases TNF-α cell content by activation of the RhoA pathway in urothelial cells [21]. Transmembrane precursors and soluble forms were stimulated by LPS in urothelial cells, and this effect was abolished by THX-B, suggesting a paracrine action of LPS-stimulated proNGF on receptor p75^NTR^, as previously reported in retinal and urothelial cells [21,42]. On the other hand, iNOS content and NO secretion increases in the presence of LPS were unaffected by THX-B, suggesting that LPS receptor TLR4 alone is responsible for this increase. In accordance, we previously reported that proNGF has a weak inhibitory action on NO secretion by urothelial cells in vitro [21]. LPS-elicited NO secretion by urothelial cells was abolished by inhibition of protein kinase C (PKC), a pathway unrelated to p75^NTR^ and linked to TLR4 [15,16]. Secretion of TNF-α by SMC in vitro was already reported to be unaffected by proNGF, confirming the present observation [21].

Intracellular second messengers triggered by LPS are mainly ERK, JNK, and p38-MAPK, while p75^NTR^ is more selective for JNK, which is activated after binding of protein TRAF6 to the intracellular portion of p75^NTR,^ leading to activation of NF-κB [27]. In UROs, an increase in P-ERK content in response to LPS was unaffected by THX-B, suggesting that this activation was carried out by LPS receptors. On the other hand, p38-MAPK was decreased by LPS and was well unaffected by THX-B, confirming that this pathway is specific for TLR4. LPS is usually associated with an increase in p38-MAPK, which translates once more the specificity of this cell type [15,39]. Finally, JNK activation was not affected by LPS, nor was TRAF6 bound to p75^NTR^ or NF-κB nuclear content, confirming that neither receptor was associated with this second messenger in urothelial cells [21]. SMCs, on the other hand, showed a pattern of activation at the opposite end of urothelial cells. While the ERK and p38MAPK levels of activation were unaffected by LPS, a strong stimulation of the JNK pathway, as well as TRAF6/p75 complexes and translation of NF-κB to the nucleus, could be seen. THX-B inhibition of this pro-inflammatory pathway once again suggests an indirect effect of LPS through the p75^NTR^ receptor in these cells. As there was no increase in proNGF or proBDNF, we can hypothesize that another proneurotrophin produced by SMC may activate p75^NTR^, or that THX-B inhibited basal levels of activated p75^NTR,^ which appeared sufficient to counteract the effect of LPS. These data also confirmed our previous results on SMC incubated in hyperglycemic medium, in which NF-κB translocation was increased by proNGF, leading to proliferation and migration [21]. Details of the exact mechanism involved remain to be elucidated.

Tight junctions are essential to epithelial barrier impermeability in many organs, and a loss in their expression correlates with increasing barrier permeability to ionic compounds and pathogen infiltration. However, in the past few years, the role of these tight junctions has been extended to that of a molecular effector in apoptotic signaling. ZO-1 deletion increases JNK-activated caspase-3-dependent apoptosis, whereas the suppression of occludin by ERK signaling has no particular effect on epithelial barrier function [43]. Here, our findings are supported by the existing literature suggesting that LPS activation of TLR4 downregulates ZO-1 and occludin. Antagonism of the p75^NTR^ receptor partially reintroduced occludin expression. In patients with interstitial cystitis and painful bladder syndrome, the urothelium presents a similar decreased expression of occludin and ZO-1 [43]. ProNGF, through binding p75^NTR,^ increases secretion of TNF-α, leading to a decrease in occludin expression [21]. The complexity in understanding how occludin is controlled by LPS comes from the presence of three receptors, p75^NTR^, TLR4, and TNF-α receptor, which intertwine intracellular pathways involving ERK, JNK, p38-MAPK, and NF-κB, among others. Nevertheless, from our results, given the lack of effect of THX-B on signaling pathways, we can only assume that the rescued occludin by THX-B involved binding of proNGF on p75^NTR^ after LPS stimulation and probably the subsequent decrease in TNF-α, as previously reported in vitro on SMC [21]. ZO-1 was also affected by LPS and was insensitive to the action of THX-B. This difference in the answer to LPS between occludin and ZO-1 has already been reported and was related to completely different intracellular pathways triggered by LPS [44].

Activation of JNK by LPS in the bladder wall was prevented by THX-B injection. The absence of TNF-α changes likely reflects the dilution of urothelial TNF-α within the whole-bladder protein homogenate. Still, microscopy confirmed a lower degree of inflammation as the number of immune cells in the lamina propria was dramatically decreased by THX-B treatment in LPS-treated mice. Antagonism of p75^NTR^ has been shown to decrease activation of microglial cells in the brain, preventing trafficking of inflammatory monocytes and central nervous system injuries [18]. As well, the increase in bladder wall thickness resulting from inflammation is reversed by THX-B treatment. Regarding the contractile properties of the bladder wall, THX-B did not affect the effect of LPS. This could be that the duration of infection was not long enough to trigger significant changes in bladder contractile proteins. Similarly, we carried out a voiding spot assay to examine the voiding pattern of treated animals. There were no changes observed after 24 h in terms of volume of urine and number of spots. In accordance, in a previous in vitro study, proNGF after 24 h did not affect myosin or smoothelin-phenotype-shift markers of SMC-despite enhanced SMC proliferation and migration [21].

Importantly, it has been noted that elevated intravesical pressure in bacterial cystitis can contribute to loss of bladder resistance over time and, by extension, increase host susceptibility to recurrent bacterial and chronic cystitis. Elevated intravesical pressure mainly arises after changes in detrusor morphology (hypertrophy and irreversible fibrosis), resulting in reduced tissue blood perfusion and low bladder compliance [45]. While the mechanisms regulating hypertrophic and fibrotic responses in smooth muscle insults are still largely unclear, the NF-κB and JNK pathways have been shown to enhance SMCs enlargement, proliferation, and bladder collagen I production [46]. Remarkably, we found similar activation of JNK in detrusor SMCs in contact with LPS that could be prevented by p75^NTR^ antagonism, suggesting the presence of an inflammatory overlap. Identifying similar increases in TRAF6 recruitment by p75^NTR^ and NF-κB translocation, both subsequently reduced by p75^NTR^ antagonism, confirmed that TRAF6 is a key effector of this overlap.

Our study presents THX-B as a new means to treat or prevent bacterial inflammation in the female bladder. Given its high specificity to p75^NTR^, off-target side-effects are non-existent, limiting its nocivity. In accordance, in several animal models of overactive bladder, diabetic retinopathy, and retinitis pigmentosa, chronic injection of THX-B proved that this component to be easily delivered to the target tissue, is very well tolerated, and with very few side effects [21,47]. On the other hand, some limitations arise when looking at the in vitro setting. Only one dose of THX-B was used in vitro and in vivo, as previously one in studies in the field. Dose–response curves could have provided a better insight into THX-B efficiency in the treatment of LPS infection. Also, we tested only one type of LPS (O55:B5). Many different sort exists (O111:B4, O127:B8, or O26:B6) which reflect the diversity of *E. coli* strains. On the other hand, signaling pathways involved might be sex-specific and therefore different between male and female bladders. Further studies need to be conducted in vitro and in vivo on the male bladder. Another limitation concerns our in vivo assay. Insertion of the urethral catheter, even if carried out in the most sterile conditions, could by itself lead to irritation or inflammation of the urethra and bladder wall. The addition of another group of animals with no catheter insertion would help determine more precisely the origin of the bladder inflammation observed, whether it originates from the LPS or the procedure, or both.

## 5. Conclusions

In conclusion, this paper showed that THX-B, an inhibitor of pro-inflammatory receptor p75^NTR^, can decrease LPS-activated inflammatory processes in the bladder tissue, in vitro and in vivo, by acting on both urothelial and smooth muscle cells. p75^NTR^ antagonism might constitute a new pharmacological approach to treating or preventing bladder infections caused by *E. coli*.

## Data Availability

The raw data supporting the conclusions of this article will be made available by the authors on request.

## Abbreviations

The following abbreviations are used in this manuscript:

CCAC	Canadian Council for Animal Care
DMEM	Dulbecco’s Modified Eagle Medium
EFS	Electrical Field Stimulation
ERK	Extracellular Signal-Regulated Kinase
FBS	Fetal Bovine Serum
H&E	Hematoxylin and Eosin
IC/BPS	Interstitial Cystitis or Bladder Pain Syndrome
iNOS	Inducible Nitric Oxide Synthase
JNK	c-Jun N-terminal Kinase
LPS	Lipopolysaccharide
NEDD	N-(1-Naphthyl)ethylenediamine dihydrochloride
NF-κB	Nuclear Factor Kappa B
NO	Nitric Oxide
p38MAPK	p38 Mitogen-Activated Protein Kinase
p75^NTR^	p75 Neurotrophin Receptor
PBS	Phosphate-Buffered Saline
PKC	Protein Kinase C
proBDNF	Proform of Brain-Derived Neurotrophic Factor
proNGF	Proform of the Nerve Growth Factor
SD	Sprague Dawley
SMC	Smooth Muscle Cell
TBST	Tris-Buffered Saline with Tween 20
THX-B	pharmacological antagonist of p75^NTR^
TLR4	Toll-Like Receptor 4
TNF-α	Tumor Necrosis Factor alpha
TRAF6	Tumor Necrosis Factor Receptor-Associated Factor 6
UTI	Urinary Tract Infection
Y27623	Rho inhibitor 4-((1R)-1-aminoethyl)-N-pyridin-4-ylcyclohexane-1-carboxamide
ZO-1	Zonula Occludens 1
